# From inactivation to intervention: X chromosome silencing in disease pathogenesis and emerging therapeutic strategies

**DOI:** 10.1016/j.gendis.2025.101964

**Published:** 2025-12-05

**Authors:** Yuan Fu, Xuling Tan, Lixia Qin, Chunyu Wang

**Affiliations:** a Department of Medical Genetics, The Second Xiangya Hospital of Central South University, Changsha, Hunan 41001, China; b Department of Neurology, The Second Xiangya Hospital of Central South University, Changsha, Hunan 41001, China; c Key Laboratory of Hunan Province in Neurodegenerative Disorders, Central South University, Changsha, Hunan 41001, China; d Clinical Medical Research Center for Stroke Prevention and Treatment of Hunan Province, Department of Neurology, The Second Xiangya Hospital of Central South University, Changsha, Hunan 41001, China; e Department of Medical Genetics, Hunan Province Clinical Medical Research Center for Genetic Birth Defects and Rare Diseases, The Second Xiangya Hospital of Central South University, Changsha, Hunan 41001, China

**Keywords:** Detection of X chromosome inactivation, Mechanisms, The inactive X chromosome reactivation, Therapeutic strategies, X chromosome inactivation

## Abstract

X chromosome inactivation (XCI) is a crucial epigenetic mechanism that balances X-linked gene expression in females via random silencing of one X chromosome. Skewed XCI—non-random inactivation favoring one allele—impacts disease penetrance in X-linked disorders. In heterozygous females, phenotypic severity correlates with XCI skewing degree. Accurate XCI quantification is critical for predicting clinical variability and improving risk assessment in X-linked mutation carriers. The X inactivation-specific transcript (*Xist*) gene drives XCI initiation through its long non-coding RNA (lncRNA) that recruits polycomb repressive complexes 2 (PRC2) to establish stable heterochromatin. Bracingly, emerging therapies leveraging XCI reactivation (*e.g.*, *Xist* RNA inhibition, *Xist* RNA epigenetic modification) show preclinical potential to rescue silenced alleles, advancing treatment strategies for X-linked diseases. This review synthesizes XCI mechanisms, current skewing detection methods, and therapeutic developments, providing a roadmap for clinical translation of XCI-targeted interventions.

## Introduction

In 1949, cytogeneticists Murray Barr and Ewart Bertram made a seminal discovery using Feulgen staining: they identified a distinct chromatin body adjacent to the nuclear envelope in female cat neurons, which was conspicuously absent in male specimens.^1^ This dense, drumstick-shaped structure, subsequently termed the Barr body, became recognized as the cytological manifestation of X chromosome inactivation (XCI). By 1960, Susumu Ohno proposed that mammals exhibit two distinct forms of the X chromosome: one resembling transcriptional activity of an autosome, and the other adopting a condensed, heterochromatic state, corresponding to the Barr body.^2^ This foundational work culminated in Mary F. Lyon’s groundbreaking 1961 hypothesis, which established three pivotal principles: i) random inactivation of one X chromosome in female somatic cells during embryogenesis, ii) irreversible heterochromatinization of the inactivated X, and iii) clonal propagation of this epigenetic state, thereby achieving dosage equilibrium between XY males and XX females.^3^

XCI is a critical mechanism in female mammals for balancing the dosage of X-linked genes, ensuring equivalent gene expression between males and females through randomly silencing one X chromosome during early embryogenesis.^4^ This process is regulated by the long non-coding RNA (lncRNA) gene X inactivation-specific transcript (*Xist*).^5^ As the master regulator of XCI, *Xist* resides within the X-inactivation center (XIC), making its functional characterization essential for deciphering XCI mechanisms.

Originally recognized for its role in dosage compensation, XCI is now understood to have broader biological implications. Skewed XCI, where one X chromosome is preferentially inactivated, contributes to phenotypic variability in X-linked disorders, correlating with disease severity. Consequently, female patients with the same mutation may display different disease severities due to varying degrees of X chromosome skewing.^6^ It was previously believed that once XCI is established, deletion of *Xist* or the entire XIC in somatic cells would not lead to observable reactivation of the inactive X chromosome (Xi).^7^ However, with technological advancements, it has been shown that conditional deletion of *Xist* can result in the reactivation of individual genes on the Xi.^8^ These insights have propelled XCI modulation into therapeutic focus, with two emerging strategies showing promise: i) Reactivation of the Xi to restore gene expression, and ii) Correction of skewed XCI patterns to mitigate clinical heterogeneity.

Recent studies on skewed XCI and its association with X-linked phenotypes are advancing. While most research on XCI reactivation as a potential therapeutic strategy has focused on Rett syndrome (RTT), some studies have also investigated other conditions, such as X-linked sideroblastic anemia (XLSA). However, considerable challenges remain before these findings can be applied in clinical practice. First and foremost, it is crucial to elucidate the mechanisms governing the initiation and maintenance of XCI in humans, as this will provide a robust foundation for future therapeutic advancements. Additionally, accurately quantifying the skewing ratio of XCI is essential for developing personalized treatment strategies.

This review systematically examines four critical domains: i) Molecular mechanisms underlying XCI; ii) Impact of XCI skewing on phenotypic variability in X-linked diseases; iii) Technological evolution in XCI assessment; iv) Translational applications of XCI modulation.

## Mechanism of inactivation of the X chromosome: *Xist* locus

In mice and humans, the XIC is a genomic region on the X chromosome responsible for initiating XCI.^9^^,^^10^ In 1991, the *Xist* gene, a novel X-linked gene exclusively expressed from the Xi, was identified within the XIC as being central to XCI.^5^ Subsequent comparative analyses demonstrated that *Xist* is evolutionarily conserved in both sequence composition and genomic organization across mammalian species.^11^^,^^12^

**Figure 1:**
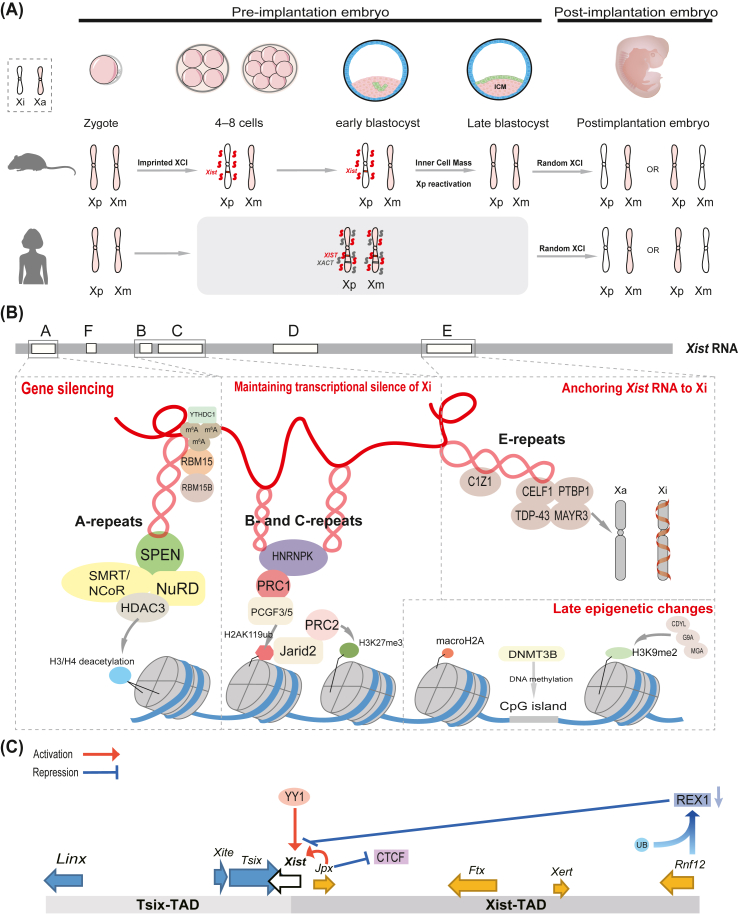
Key regulatory genes and elements in X chromosome inactivation (XCI). **(A)** Differences in the initiation of XCI between mice and humans. In humans, both X chromosomes undergo transcriptional dampening during the early pre-implantation stage, with X-inactive specific transcript (*XIST*) expressed from both X chromosomes to ensure dosage compensation. XACT transiently co-expresses with *XIST*, but does not regulate *XIST* or X-linked gene expression. In mice, imprinted XCI (iXCI) silences the paternal X chromosome (Xp) early, beginning at the 4-cell stage. During the late blastocyst stage, Xp is reactivated in the inner cell mass (ICM). Random X-chromosome inactivation (rXCI) occurs at the late blastocyst stage in both mice and humans. **(B)** Functional domains of *Xist* RNA and late epigenetic changes. Repeat A initiates transcriptional silencing by interacting with the SPlit ENds (SPEN) protein, which further recruits chromatin-modifying complexes to promote histone H3/H4 deacetylation. It also recruits repressor complexes, including nuclear receptor corepressor (NCoR), silencing mediator of retinoic acid and thyroid hormone receptor (SMRT), and nucleosome remodeling deacetylase (NuRD), to the inactive X chromosome (Xi), facilitating histone deacetylation via histone deacetylase 3 (HDAC3). Additionally, repeat A recruits SPOC-containing proteins, such as RNA-binding motif proteins 15 (RBM15) and 15B (RBM15B), which mediate gene silencing through N6-methyladenosine (m^6^A) RNA methylation. m^6^A modifications in the Xist repeat A region are recognized by the m^6^A reader YTH domain containing 1 (YTHDC1), contributing to transcriptional silencing. Repeats B and C maintain Xi in its inactive state by recruiting polycomb repressive complexes 1 (PRC1) and 2 (PRC2). PRC1 mediates the silencing of CpG island (CGI)-associated genes through histone H2A lysine 119 mono-ubiquitination (H2AK119ub1), while PRC2 targets a distinct or partially overlapping set of genes via histone H3 lysine 27 trimethylation (H3K27me3). HNRNPK recruits the PCGF3/5-PRC1 complex, which catalyzes H2A lysine 119 ubiquitination (H2AK119ub), marking silenced chromatin regions. Jarid2, a cofactor of PRC2, recognizes H2AK119ub and promotes PRC2 recruitment to the Xi, further reinforcing heterochromatin formation. The Repeat E module anchors *Xist* RNA to the Xi, crucial for maintaining XCI. CDKN1A-interacting zinc finger protein 1 (CIZ1) aids *Xis*t’s association with the nuclear matrix in a cell lineage-specific manner. Four proteins, PTBP1, MATR3, TDP-43, and CELF1, interact with Repeat E, forming a CIZ1-independent complex that stabilizes *Xist* RNA after transcriptional silencing by Repeat A and PcG recruitment via Repeat B/C. The roles of Repeats D and F are less well defined, though Repeat F may influence *Xist* RNA stability. Late-stage epigenetic modifications include macroH2A enrichment on the Xi, DNA methylation of CpG islands by DNA (cytosine-5)-methyltransferase 3B (DNMT3B), and histone H3 lysine 9 dimethylation (H3K9me2). H3K9me2 recruits the chromodomain Y-like (CDYL)–G9A–MAX gene-associated (MGA) complex, which may propagate H3K9me2 across the Xi. **(C)** Regulatory landscape of the X inactivation center (XIC). The XIC orchestrates XCI, with the *Xist* gene acting as the central regulator. *Xist* expression is negatively controlled by the antisense transcript X-inactive specific transcript antisense (*Tsix*), which overlaps *Xist* and is transcribed in the opposite direction. *Tsix* is located within the *Tsix* topologically associating domain (TAD), while *Xist* resides in the adjacent *Xist* TAD. The *Xist* TAD contains several positive regulators of *Xist*, including the non-coding RNAs *Jpx* and *Ftx*, the RING finger protein 12 gene (*Rnf12*), and the enhancer-associated transcript *Xert*. *Linx*, a long non-coding RNA, acts as a distal cis-repressor of *Xist*, and the *Xite* enhancer drives *Tsix* transcription. *Jpx* RNA activates *Xist* by displacing CCCTC-binding factor (CTCF), an RNA-binding protein that represses *Xist* transcription. During differentiation, the degradation of the pluripotency factor REX1 (also known as zinc finger protein 42, ZFP42) relieves repression of *Xist*, enabling its upregulation. Additionally, the transcription factor Yin-Yang 1 (YY1) competes with REX1 for binding to the *Xist* 5′ region, thereby activating the *Xist* promoter. Xa, active X chromosome; Xi, inactive X chromosome; Xp, paternal X chromosome; Xm, maternal X chromosome.

Although XCI is conserved in female mammals, its early silencing mechanisms exhibit marked divergence between rodents and primates.^13^ In humans, both X chromosomes undergo transcriptional dampening rather than complete inactivation during the early pre-implantation embryo stage. *XIST* is expressed from both X chromosomes to ensure early dosage compensation at the blastocyst stage.^14^ The lncRNA X active-specific transcript (XACT) transiently co-expresses with *XIST*.^15^ XACT is an X-linked transcriptional unit spanning over 250 kb, located approximately 50 Mb distal to *XIST*. It is identified in humans and conserved among great apes, but absent in lesser apes and more distantly related species.^16^ However, recent studies using naive human embryonic stem cells indicate that XACT does not significantly regulate *XIST* expression, localization, or function, nor does it influence X-linked gene expression during the pre-implantation stage, suggesting XACT is dispensable for X chromosome transcriptional regulation at this stage.^17^ Random X-chromosome inactivation (rXCI) occurs only at the late blastocyst stage.^13^ In mice, imprinted X-chromosome inactivation (iXCI) occurs, specifically silencing the paternal X chromosome (Xp).^18^ After fertilization, both maternal and paternal X chromosomes are initially active. iXCI is initiated at the four-cell stage, and by embryonic day 3.5 (E3.5), all embryonic cells have an inactive Xp.^19^ Xp remains inactive in extraembryonic tissues but is reactivated in the inner cell mass during the blastocyst stage. Similar to humans, rXCI occurs at the late blastocyst stage (Fig. 1A).^20^^,^^21^

Given that it is coated by *Xist* RNA, the X chromosome undergoes gene silencing. Most research investigating the mechanisms driving XCI has relied on dissecting the function of *Xist* RNA itself. In mice*, Xist* RNA is structurally organized into multiple Repeat modules (A–F),^11^ which act as functional modules during XCI (Fig. 1B).^22^ These modules interact with chromatin modifiers and spatial organization factors, playing essential roles in establishing and maintaining the silenced state of the Xi.

The Repeat A module (nucleotides 335–700 of *Xist*), the most evolutionarily conserved RNA domain, is indispensable for Xi-wide transcriptional silencing of nearly all genes on the Xi. Interacting with Repeat A via its RNA recognition motifs (RRMs),^23^ SPlit ENds (SPEN), a ribonucleoprotein (RBP) containing an SPEN paralogue/orthologue C-terminal (SPOC) domain, is recruited to active promoters and enhancers across the X chromosome, initiating gene silencing,^24^ and further mediates the recruitment of chromatin-modifying complexes to promote histone H3/H4 deacetylation and hinder RNA polymerase II (Pol II) loading.^25^^,^^26^ Meanwhile, the SPOC domain of SPEN is involved in the recruitment of several repressor complex members of NR corepressor (NCoR), the silencing mediator of retinoic acid or thyroid hormone receptor (SMRT), and nucleosome remodeling deacetylase (NuRD) complex.^24^ SPEN potentially activates part of the histone deacetylase 3 (HDAC3) population pre-bound to the Xi enhancer by delivering NCoR/SMRT complex members, leading to histone deacetylation and facilitating histone 3 lysine 4 monomethylation (H3K4me1) demethylation.^27^ Additionally, Repeat A recruits other SPOC-containing proteins, such as RNA-binding motif protein 15 (RBM15) and 15B (RBM15B), which facilitate gene silencing through N^6^-methyladenosine (m^6^A) RNA methylation.^28^ The m^6^A is deposited along the m^6^A hotspot in the downstream *Xist* transcript located in the Repeat A region. This modification is subsequently recognized and bound by the m^6^A reader YTH domain-containing 1 (YTHDC1).^29^^,^^30^

The Repeat B module (nucleotides ∼2850–3050) and the Repeat C module (nucleotides ∼2850–3050) are responsible for maintaining the transcriptionally silent state of the Xi. After the initial establishment of silencing by the Repeat A/SPEN module, the B/C Repeat modules recruit polycomb group (PcG) protein complexes to the Xi.^31^ Among these complexes, polycomb repressive complexes 1 (PRC1) and 2 (PRC2) are essential in *Xist*-mediated chromosomal silencing.^32^ PRC1 and PRC2 function in an independent yet complementary manner during the maintenance phase of XCI, with PRC1 mediating the silencing of CpG island (CGI)-associated genes via histone H2A lysine 119 mono-ubiquitination (H2AK119ub1), and PRC2 targeting a distinct or partially overlapping set of genes through histone H3 lysine 27 trimethylation (H3K27me3), thereby cooperatively ensuring stable silencing of the Xi in extra-embryonic tissues.^33^ The B/C Repeat modules, enriched in cytosine residues, are recognized by heterogeneous nuclear ribonucleoprotein K (HNRNPK).^34^ HNRNPK recruits the PCGF3/5-PRC1 complex, which catalyzes H2A lysine 119 ubiquitination (H2AK119ub), a chromatin modification marking silenced regions.^32^^,^^35^ Additionally, Jarid2, a PRC2 cofactor, recognizes H2AK119ub and facilitates PRC2 recruitment to the Xi, further reinforcing heterochromatin formation.^36^

The Repeat E module (nucleotides ∼10,275–11,400) mediates interactions with multiple proteins to anchor *Xist* RNA to the Xi, playing a critical role in maintaining XCI. CDKN1A-interacting zinc finger protein 1 (CIZ1) facilitates the association of *Xist* with the nuclear matrix in a cell lineage-specific manner.^37^^,^^38^ Additionally, four proteins, polypyrimidine tract-binding protein 1 (PTBP1), Matrin 3 (MATR3), TAR DNA-binding protein 43 (TDP-43), and CUGBP Elav-like family member 1 (CELF1), directly interact with the Repeat E module, forming a CIZ1-independent complex. This complex stabilizes *Xist* RNA coating on the Xi, after the transcriptional silencing initiated by the Repeat A module and PcG recruitment mediated by the Repeat B/C modules.^39^

The roles of the Repeat D and F modules are less well understood. Genetic ablation of the Repeat D module appears to have no discernible impact on *Xist* RNA coating of the Xi or PcG protein recruitment.^35^ In contrast, deletion of the Repeat F module disrupts *Xist* RNA stability, leading to failure in transcriptional silencing.^35^^,^^40^^,^^41^

The Repeat elements within the human *XIST* gene are relatively conserved compared with their counterparts in mice, albeit with notable differences. In humans, the B Repeat is split into two distinct segments, termed Bh and B. Additionally, the extent of Repeat expansion varies between species. Notably, the C Repeat is substantially expanded in mice, a feature that appears to be rodent-specific, whereas in humans it is reduced to a single monomeric unit. In contrast, the D Repeat exhibits a marked expansion in human *XIST* but predominantly consists of truncated monomers in the rodent lineage.^42^ Despite these sequence divergences, the higher-order folding of *Xist* RNA is conserved between mice and humans, partitioned into five major structural modules: Repeat A, F, B/C/D, E, and exon 6/7. The E Repeat is located at the 5 end of exon 6 of *XIST*/*Xist* and at the 5 end of exon 7 of *XIST*/*Xist,* respectively; all other Repeats are contained within the large first exon of *XIST*/*Xist*.^30^^,^^43^

Mouse models have long served as the primary system for studying XCI in mammals and have been instrumental in elucidating the role of *Xist* in gene silencing. During XCI, specific Repeat modules and structural motifs in *Xist* RNA from mouse embryonic stem cells recruit chromatin remodelers and transcriptional repressors, driving chromatin compaction and gene silencing.^44^^,^^45^ During *Xist* transcription, *Xist* RNA accumulates at discrete “entry sites” on the X chromosome destined for inactivation, subsequently spreading to distal genomic regions to form a contiguous RNA coating.^46^ This RNA-mediated coating coincides with chromatin structural reorganization, marking the onset of XCI.

During the maintenance phase of XCI, late-stage epigenetic modifications include the specific enrichment of the histone variant macroH2A on the Xi,^47^ DNA methylation of CpG islands by DNA (cytosine-5)-methyltransferase 3B (DNMT3B),^48^ and histone H3 lysine 9 dimethylation (H3K9me2). H3K9me2 facilitates the recruitment of the chromodomain Y-like (CDYL)–G9A–MAX gene-associated (MGA) complex, which may, in turn, promote the propagation of H3K9me2 across the Xi.^49^ However, the precise roles of these late-stage epigenetic modifications in the maintenance of XCI, as well as their dependency on *XIST* RNA, remain unclear.

Ultimately, the assembly of *Xist*-scaffolded protein complexes at the Xi locus initiates a cascade of events—silencing initiation, spreading, and maintenance—culminating in the establishment of a repressive chromatin state and three-dimensional chromosomal restructuring.

## Regulatory networks controlling *Xist* expression

Up-regulation of *Xist* is essential for initiating XCI. The *Xist* antisense non-coding transcription unit *Tsix* is the first and most prominent negative regulator of *Xist*.^50^ *Tsix* suppresses ectopic expression of *Xist* on the maternal X chromosome; mutations in *Tsix* disrupt this suppression, leading to abnormal *Xist* up-regulation. Chromosome conformation capture studies of the XIC show that this locus is divided into at least two topologically associating domains (TADs): the *Xist* TAD and *Tsix* TAD (Fig. 1C).^51^ Within these domains, intra-domain chromatin interactions are more frequent than inter-domain contacts.^52^ The *Xist* promoter is located within the *Xist* TAD, which also contains several positive regulatory elements, including the proximal non-coding genes *Jpx* and *Ftx*, the ring finger protein 12 (RNF12) gene (*Rnf12*),^53^^,^^54^ and the *Xist*-enhancing regulatory transcript (*Xert*), all of which are up-regulated during cellular differentiation. In contrast, the *Tsix* TAD contains the Xite enhancer for *Tsix*^55^ and the long non-coding RNA *Linx*, which acts as a distal cis-acting repressor of *Xist*.^10^

*Xist* and *Tsix* are localized at the boundary between two TADs.^55^ *Jpx*, located 10 kb upstream of the *Xist* locus, acts as a trans-activator of *Xist* through both cis- and trans-acting mechanisms. It activates *Xist* by displacing CCCTC-binding factor (CTCF), an RNA-binding protein that represses *Xist* transcription.^56^ *Ftx*, another upstream non-coding RNA locus, acts as a cis-acting regulatory element. In human cellular models, a 453 kb deletion overlapping *Jpx* and *Ftx* causes skewed *Xist* expression on the intact X chromosome.^57^ The *Xert* locus, approximately 200 kb upstream of *Xist*, is co-up-regulated with *Jpx* and *Ftx* during cellular differentiation. The *Xert* promoter (*XertP*) and *Xert* enhancer cluster (*XertE*) enhance *Xist* transcriptional activity and also elevate *Ftx* expression through cis-regulatory interactions.^53^

In addition to these non-coding elements, the *Xist* TAD also harbors the *Rnf12* gene, which encodes an E3 ubiquitin ligase. RNF12 acts as a dose-dependent trans-activator of *Xist*,^58^ promoting the proteasomal degradation of the pluripotency transcription factor REX1 (also known as zinc finger protein 42 or ZFP42). During cellular differentiation, REX1 degradation facilitates *Xist* up-regulation and initiates XCI.^59^ RNF12 also synergistically interacts with the cis-regulatory regions of *Jpx* and *Ftx.*^60^ Furthermore, the autosomal trans-activator Yin Yang 1 (YY1) competes with REX1 for binding to the 5′ region of *Xist*, thereby activating its promoter.^61^ Recent studies have identified YY1 as a key gene-intrinsic regulatory factor in XCI, whereby its binding at promoters and enhancers of slow-silencing genes serves to delay the progression of *Xist*-mediated gene silencing.^62^

The *Tsix* transcript originates ∼15 kb downstream of *Xist*, partially overlapping its locus and transcribed in the antisense orientation. The long non-coding RNA *Linx*, located ∼150 kb upstream of the *Xite* enhancer, facilitates *Xist* transcriptional activation and XCI initiation through its promoter *LinxP*.^63^^,^^64^

In contrast to murine *Tsix,* human *TSIX* does not fully overlap with the *XIST* locus and is co-expressed with *XIST* from the Xi, challenging its proposed role as a transcriptional repressor of *XIST*.^65^^,^^66^ Consistent with this, the *TSIX* TAD, aside from the *LinxP* element, shows limited conservation compared with the highly conserved *XIST* TAD.^64^

In humans, *JPX* and *FTX* are conserved within the same TAD as *XIST*, yet exhibit pronounced functional divergence between species that is not explained by primary sequence conservation. While the function of *FTX* is not conserved in humans, *JPX* serves as a key regulator of *XIST* expression in both humans and mice. Importantly, unlike in mice, human *XIST* expression is regulated by the transcriptional activity of *JPX* rather than the accumulation of *JPX* RNA.^54^ These findings underscore the remarkable functional plasticity of conserved lncRNA loci within the regulatory architecture of XCI.

## X chromosome inactivation and phenotypic manifestations of X-linked disorders

In humans, XCI initiates during the early stages of embryonic implantation. The selection of which X chromosome—maternal (Xm) or Xp—is to be inactivated is traditionally considered a random process, with both parental chromosomes having equal probability of being silenced.^67^ Under purely random conditions, the expected ratio of maternal to paternal XCI is 1:1 (50:50). However, deviations from this expected ratio result in skewed XCI. Extreme skewing occurs when ≥ 90% or ≥ 95% of cells inactivate the same X chromosome. In the general female population, XCI patterns exhibit considerable variability, ranging from highly skewed (0:100, where one X chromosome remains active in all cells) to balanced (50:50, where both X chromosomes are equally active across cells).^68^^,^^69^ This variability underscores the stochastic nature of XCI and its contribution to phenotypic diversity in females.^70^

Previous studies have shown a correlation between skewed XCI and phenotypic variability in female carriers of X-linked disorders. The expression of pathogenic alleles can influence the clinical manifestations in female carriers of X-linked mutations, explaining the observed variability in clinical presentations among affected individuals. Several X-linked disorders exhibit non-random XCI, such as Fabry disease, Duchenne muscular dystrophy,^71^ hemophilia, Becker muscular dystrophy, and X-linked chronic granulomatous disease (Table 1). In this section, we focus on RTT, a well-characterized X-linked disorder, to illustrate how XCI skewing impacts disease phenotypes.

**Table 1:** Association of X chromosome inactivation (XCI) skewing and phenotypes in X-linked disorders.

X-linked disease	OMIM	Gene	Study population	Detection method	Phenotype and X-inactivation correlation	Reference
Aicardi syndrome	304050	*Xp22*	35 females	HUMARA assay	Non-random XCI is associated with a high neurological composite severity score	72
Angioma serpiginosum	300652	*PORCN*	A three-generation family	HUMARA assay	Variability in severity is explained by functional mosaicism in females because of XCI	73
ATRX syndrome	301040	*ATRX*	A 4-year-old girl	The methylation patterns at the *AR* and at the *FMR1* loci	The active chromosome carries a heterozygous mutation in the ATRX gene	74
Becker muscular dystrophy	300376	*DMD*	36 carriers from 11 families	HUMARA assay	The onset of symptoms in the carriers is related to a skewed XCI	6
Conradi-Hünermann-Happle syndrome	302960	*EBP*	13 female patients belonging to 9 Spanish families	HUMARA assay	The skewed X chromosome inactivation may explain the clinical phenotype in some familial cases	75
Congenital nystagmus	310700	*FRMD7*	A four-generation family with 5 affected members	HUMARA assay	The presence of the disease in one affected girl is due to the preferential activation of the X chromosome bearing the pathogenic variant	76
Dyskeratosis congenita	305000	*DKC1*	7 subjects in 5 families	HUMARA assay	All female carriers had similarly skewed X-inactivation in multiple tissue types, regardless of phenotype	77
Duchenne muscular dystrophy	310200	*DMD*	54 patients	HUMARA assay	Clinical manifestations in carriers are associated with non-random patterns of XCI	78
Fabry disease	301500	*GLA*	56 consecutive female patients	HUMARA assay	Skewed XCI in female patients affects the disease course based on the predominantly expressed allele	79
			12 female patients		XCI is not a main factor in the phenotype variability of Fabry disease manifestation in heterozygous females	80
Fragile-X-associated tremor/ataxia syndrome	300623	*FMR1*	10 women patients and 21 without disease	*FMR1* CGG repeats and methylation status and HUMARA assay	The skewed XCI of the normal FMR1 allele may be a risk factor for the development of this disease	81
Hemophilia A	306700	*F8*	215 carriers	HUMARA assay	Skewed XCI may contribute to the low expression of clotting factor levels and bleeding symptoms	82
Hemophilia B	306900	*F9*	Twin girls	Polymorphic markers, SNP	Skewed inactivation causes severe and mild phenotypes	83
Hypoxanthine-guanine phosphoribosyltransferase deficiency	308000	*HPRT1*	109 women belonging to 31 families	HUMARA assay	There is a correlation between skewed XCI and the severity of the phenotype	84
Lowe syndrome	300179	*OCRL*	12 subjects belonging to a large four-generation family	HUMARA assay	Lowe syndrome may manifest the full phenotype in females because of the skewed X inactivation	85
Menkes disease	309400	*ATP7A*	A 6.5-month-old girl	HUMARA assay	The patient showed a severe phenotype because of the inactivation of the normal X chromosome in all the cells	86
			10 members of a family		All six female heterozygotes show skewed X-inactivation, with preferential silencing of the mutant X chromosome	87
Microphthalmia with linear skin defects	309801	*HCCS*	11 females	HUMARA assay	The degree of skewed X-inactivation causes clinical variability	88
Mucopolysaccharidosis type II	309900	*IDS*	The second child of healthy, non-consanguineous parents	HUMARA assay	A skewed inactivation silencing preferentially the X chromosome carrying the wild-type IDS gene should be responsible for the disease manifestation	89
Pelizaeus-Merzbacher disease	312080	*PLP1*	A girl with healthy parents and 2 elder brothers	HUMARA assay	The patient is severely symptomatic due to the unfavorable X-inactivation pattern	90
Pyruvate dehydrogenase complex deficiency	312170	*PDHA1*	A female monozygotic twin pair	HUMARA assay	X-chromosome inactivation may influence the phenotypic expression of the same mutation in heterozygous females	91
Simpson-Golabi-Behmel syndrome type 1	312870	*GPC3*	3 siblings	HUMARA assay and the analysis of the CCG repeats of the Fragile XE (*FRAXE*) gene	These X-inactivation studies with peripheral blood DNA specimens can provide explanations for the phenotypic features in the two female subjects	92
X-linked adrenoleukodystrophy	300100	*ABCD1*	5 heterozygous females	HUMARA assay	Skewed XCI in favor of the mutant ABCD1 allele would be associated with the manifestation of heterozygous symptoms	93
X-linked Alport syndrome	303630	*COL4A5*	A 23-year-old female and her mother	HUMARA assay	Non-random X-chromosome inactivation with a normal allele affects the phenotype of heterozygous individuals	94
			74 adult female patients		Genotype and XCI are factors associated with severity in females with this disease	95
X-linked chronic granulomatous disease	306400	*CYBB*	The female witness and her one son and one daughter	HUMARA assay	Skewed X-inactivation causes disease in carriers	96
X-linked hypohidrotic ectodermal dysplasia	305100	*EDA*	The female witness and her son	RT-PCR	XCI explained patches of abnormal skin	97
X-linked protoporphyria	300752	*ALAS2*	11 heterozygous females	HUMARA assay and ZMYM type 3 short tandem repeat polymorphisms	The Xi pattern directly influences the penetrance and the severity of the phenotype in heterozygous females	98
X-linked sideroblastic anemia	300751	*ALAS2*	3 women from a family	HUMARA assay	Their differing clinical courses can be explained by the X-inactivation patterns of granulocytes and bone marrow cells	99

Note: HUMARA, human androgen receptor; SNP, single-nucleotide polymorphism; *FMR1*, fragile X messenger ribonucleoprotein 1.

**Figure 2:**
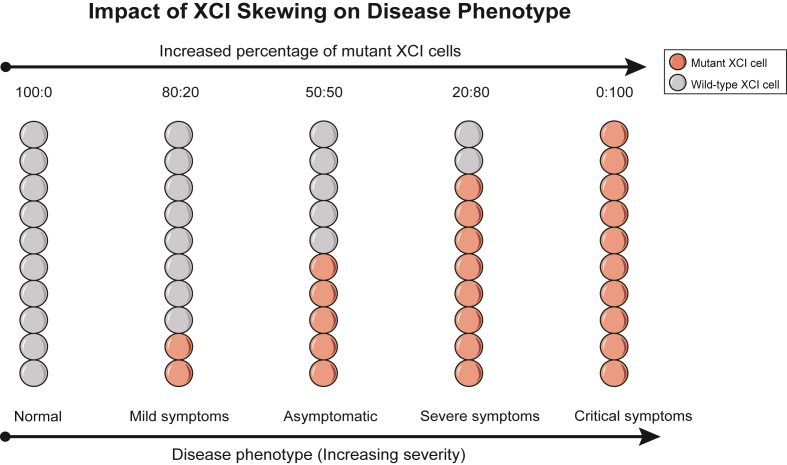
Impact of X chromosome inactivation (XCI) skewing on disease phenotype. Under random XCI (WT:Mut = 50:50), cells exhibit an equal expression of both wild-type and mutant alleles, leading to an asymptomatic phenotype. In non-random XCI, the degree of skewing towards the mutant allele directly correlates with the increasing severity of clinical manifestations. At WT:Mut = 80:20, the preferential inactivation of the mutant allele results in mild clinical symptoms. At WT:Mut = 20:80, inactivation of the wild-type allele predominates, leading to severe functional deficits. At WT:Mut = 0:100, complete inactivation of the wild-type allele causes profound disability and life-threatening complications. Conversely, when XCI is fully skewed towards the wild-type allele (WT:Mut = 100:0), inactivation of the mutant allele restores wild-type expression, resulting in a phenotypically normal outcome. WT:Mut: X-inactivation ratio (Wild-type to Mutant).

RTT is a rare neurodevelopmental disorder that predominantly affects females.^100^ Affected individuals typically experience normal development during the first 6–18 months of life, followed by developmental plateauing, regression of acquired speech and motor skills, stereotypic hand movements, gait abnormalities, and aberrant respiratory patterns. As the disorder progresses, individuals often experience severe intellectual disability, epilepsy, and profound impairments in social and communicative abilities. RTT is caused by mutations in the X-linked methyl-CpG-binding protein 2 (*MECP2*) gene, which encodes a transcriptional regulator critical for brain development.^101^ Approximately 90%–95% of classic RTT cases and 70% of atypical cases harbor *MECP2* mutations. As *MECP2* is subject to XCI, phenotypic variability in female carriers is heavily influenced by XCI skewing. In females with a heterozygous *MECP2* mutation, random XCI theoretically results in 50% of cells expressing the wild-type allele and 50% expressing the mutant allele. However, skewed XCI, where the mutant *MECP2* allele is preferentially inactivated in most cells, can reduce clinical severity or even lead to asymptomatic carrier status. Conversely, preferential inactivation of the wild-type allele exacerbates disease manifestations. Thus, the degree of XCI skewing directly correlates with the clinical heterogeneity observed in RTT (Fig. 2).

Most *de novo* mutations in the *MECP2* gene occur on the paternal allele,^102^, ^103^, ^104^ leading to exclusive transmission to female offspring. A study by Knudsen et al examined the parental origin of the Xi in RTT individuals and observed that in cases with skewed XCI, the paternally derived X chromosome (carrying the mutant *MECP2* allele) was preferentially inactivated. This finding suggests that individuals with highly skewed XCI patterns exhibit a reduced proportion of cells expressing the mutant *MECP2* allele, potentially attenuating disease severity.^105^ While the majority of RTT cases are sporadic, unaffected mothers harboring *MECP2* pathogenic variants have been reported.^106^ These carriers are likely protected by extreme XCI skewing, where the X chromosome carrying the mutant allele is predominantly silenced, minimizing its expression in critical tissues.

Despite extensive research, conflicting evidence persists regarding the correlation between XCI skewing and phenotypic severity in RTT. In a study by Huppke et al, three female carriers of *MECP2* mutations exhibited mild phenotypes despite their genetic status, with XCI skewing ratios of 84:16, 95:5, and 76:24, favoring inactivation of the mutant allele. This observation supports the hypothesis that skewed XCI attenuates disease severity by reducing mutant allele expression.^107^ Similarly, L. Villard reported an asymptomatic female carrier with extreme XCI skewing whose daughter presented with classic RTT. This illustrates that while skewed XCI in carriers does not prevent the transmission of the mutation, it can modulate phenotypic expression in offspring.^108^ Additionally, Hayley Archer et al further showed that RTT patients with *MECP2* mutations (p.R168X and p.T158M) exhibited increased clinical severity that correlated with a higher proportion of cells expressing the mutant allele. This aligns with findings that milder RTT cases are associated with greater XCI skewing toward mutant allele inactivation, though parental origin of the mutation was not determined in this study.^109^ Contrastingly, a large-scale analysis by Xiaolan Fang et al of 320 RTT cases and maternal blood samples revealed nuanced relationships between XCI skewing and clinical severity. In classic RTT patients with preferential inactivation of the maternally derived allele, a weak positive correlation (rs = 0.35, *n* = 40) was observed between XCI skewing ratios and Clinical Severity Score (CSS). However, no such correlation (rs = −0.06, *n* = 180) was found in patients with paternal allele-predominant XCI skewing.^110^ These findings highlight the complexity of XCI-phenotype interactions, which may depend on mutation origin, tissue-specific skewing, or modifier genes. The latest study of Duchenne muscular dystrophy also showed different and contradictory results than before.^111^

To reliably assess the impact of XCI skewing on phenotypic variability, future studies should focus on familial cohorts with identical *MECP2* mutations to minimize confounding effects due to mutation-specific variability. Additionally, rigorous quantification of phenotypic severity using standardized metrics, such as clinical severity scores, is essential for establishing robust phenotypic correlations. The tissue type analyzed also significantly affects findings. Most studies evaluate XCI skewing in easily accessible somatic cells (*e.g.*, leukocytes, buccal epithelial cells, urinary cells), rather than affected tissues like brain cells. However, lineage-specific XCI skewing, driven by differences in embryonic origin, may result in divergent inactivation patterns across tissues in the same individual.^112^ Age-related dynamics in XCI skewing further complicate interpretations, as skewing ratios may evolve over time.^113^ While analyzing affected tissues (*e.g.*, neurons) provides the most clinically relevant insights, this requires invasive biopsies and poses ethical concerns. Additionally, longitudinal studies are needed to address age-dependent phenotypic progression, as young patients may exhibit delayed symptom onset, which complicates cross-sectional analyses. Finally, beyond XCI skewing, other mechanisms, including germline mosaicism and epigenetic modifiers, may also contribute to phenotypic variability.^77^ A multi-factorial approach integrating genetic, epigenetic, and environmental factors is necessary to fully understand the determinants of X-linked disorders’ severity.

## Quantitative detection of XCI ratio

Since the initial observation of XCI, methodologies for detecting and quantifying XCI patterns have evolved significantly. Quantitative assessment of XCI is essential for understanding phenotypic diversity in females and evaluating disease risks associated with X-linked mutations. By determining whether XCI occurs randomly or in a skewed manner, researchers can elucidate its impact on X-linked gene expression and clinical manifestations, thereby aiding in the assessment of symptom severity in carriers of X-linked disorders. Furthermore, XCI-based therapeutic strategies—most notably Xi reactivation—are currently under active investigation. Before clinical implementation, precise quantification of XCI ratios in patients is necessary to establish baseline inactivation patterns. Therapeutic efficacy depends on achieving precise reactivation thresholds in affected cells and tissues. For instance, reactivating a small proportion (5%–10%) of Xi-linked wild-type *MECP2* alleles may be sufficient to ameliorate symptoms in RTT. Thus, accurate XCI profiling is critical for patient stratification, prediction of therapeutic responsiveness, and monitoring of treatment outcomes.

## Methylation-based analysis of XCI

During XCI, the Xi undergoes extensive DNA methylation, particularly at CpG islands, while the active X chromosome (Xa) remains largely unmethylated.^114^^,^^115^ This epigenetic divergence allows XCI status to be inferred through methylation pattern analysis at specific loci. The human androgen receptor (HUMARA) assay remains the most widely utilized method for XCI analysis. This PCR-based approach employs methylation-sensitive restriction enzymes (*e.g.*, haemophilus parainfluenzae II, HpaII) to target CpG sites in exon 1 of the X-linked Androgen Receptor (*AR*) gene.^116^ Differential methylation between Xi and Xa enables discrimination of parental alleles, where the methylated (undigested) allele corresponds to Xi.

**Figure 3:**
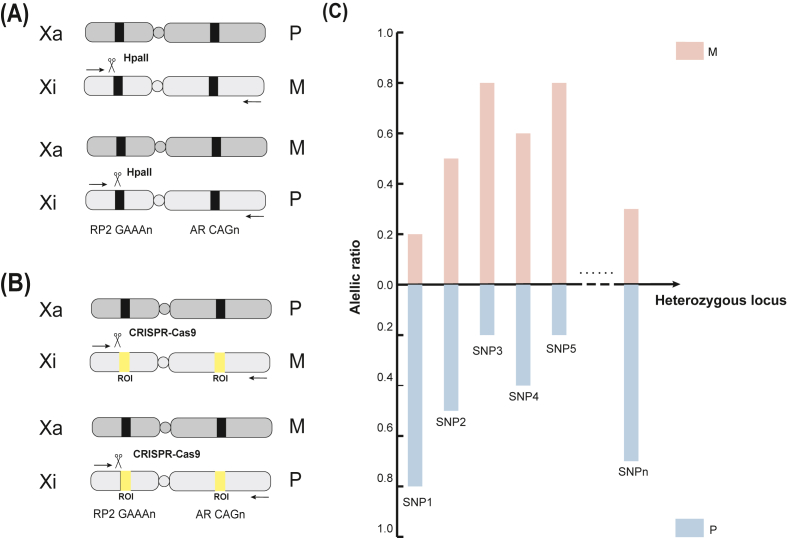
Detection methods for X chromosome inactivation (XCI) ratios. **(A)** Methylation-sensitive restriction enzyme analysis of XCI ratio using haemophilus parainfluenzae II (HpaII). The methylation-sensitive restriction enzyme HpaII was utilized to target the CAG_n_ repeat site in the *AR* gene and the GAAA_n_ repeat site in the *RP2* gene on the X chromosome. Parental alleles, distinguished by varying repeat copy numbers, were differentiated through fragment length analysis of PCR-amplified repeat sequences. The PCR products were subsequently analyzed using capillary electrophoresis with fluorescence detection, and the XCI ratio was determined based on the peak area ratio. **(B)** CRISPR-Cas9-based XCI detection with Oxford Nanopore Technologies (ONT) sequencing. The XCI-ONT method uses CRISPR-Cas9 to cut the DNA on both sides of a ∼3 kb region of interest (ROI), which spans 116 CpG sites in AR and 58 CpG sites in *RP2*. The region is then subjected to DNA sequencing, with methylation detection performed simultaneously using ONT sequencing technology. The XCI status is calculated based on the average methylation frequency of the region, followed by the calculation of the average methylation ratio between alleles for each gene. **(C)** RNA-sequencing-based XCI ratio estimation using heterozygous single-nucleotide polymorphisms (SNPs). After performing RNA-sequencing analysis on patient samples, the resulting sequence reads are aligned with the reference genome to identify heterozygous X-linked SNPs. The expression ratios of the paternal and maternal alleles at these SNP sites are then used to estimate the average XCI ratio. Xa, active X chromosome; Xi, inactive X chromosome; P, paternal allele; M, maternal allele.

In addition to *AR*, the X-linked retinitis pigmentosa 2 (*RP2*) gene has been leveraged as an alternative locus for XCI quantification due to its methylation sensitivity.^117^ Both *AR* and *RP2* contain highly polymorphic repetitive elements adjacent to CpG sites—specifically, a CAG trinucleotide Repeat in *AR* and a GAAA tetranucleotide Repeat in *RP2*—exhibiting high heterozygosity (∼0.97) and evolutionary conservation among non-human primates.^118^ Importantly, as *RP2* lacks known disease associations, it serves as a neutral alternative for XCI studies. Employing a dual *AR*/*RP2* approach improves the reliability of XCI quantification (Fig. 3A). Parental alleles are distinguished through fragment length analysis (FLA) of PCR-amplified Repeats, which vary in copy number between X chromosomes.^119^

Despite its widespread application, the HUMARA assay presents several technical limitations, including PCR artifacts (such as secondary structures or incomplete amplification, which may distort allele size distributions) and fluorescence detection bias (where fluorophore-specific signal quenching or electrophoretic anomalies may compromise haplotype resolution in FLA).

A more accurate and efficient alternative to restriction enzyme-based methods is bisulfite sequencing, which circumvents the reliance on enzymatic digestion. In this method, sodium bisulfite chemically modifies DNA by converting unmethylated cytosines to uracil, while methylated cytosines remain unchanged. This chemical conversion translates methylation differences into sequence variations, allowing amplification with methylation-specific primers designed to distinguish between methylated and bisulfite-converted (unmethylated) DNA.^120^ By using allele-specific primers and controlled PCR conditions, this approach enhances the precision and reproducibility of amplification outcomes, enabling quantitative assessment of XCI ratios.

## Advances in XCI profiling: integrating CRISPR-Cas9 and nanopore sequencing

To mitigate PCR-related biases, a novel strategy known as XCI-ONT integrates CRISPR-Cas9-mediated enrichment of *AR* and *RP2* loci with Oxford Nanopore Technologies (ONT) sequencing to simultaneously assess Repeat length and methylation status. Unlike conventional approaches, XCI-ONT directly interrogates polymorphic Repeats (*e.g.*, *AR* CAG_n_ and *RP2* GAAA_n_) and quantifies methylation at 116 CpG sites in *AR* and 58 CpG sites in *RP2* (Fig. 3B). This method significantly enhances the accuracy of XCI ratio determination by eliminating PCR amplification artifacts and providing high-resolution, long-read methylation data.^118^

## RNA-based approaches for XCI assessment

Beyond methylation-based techniques, XCI status can also be assessed at the RNA level. For instance, single-nucleotide polymorphisms (SNPs) in *Xist* RNA have been utilized to distinguish between random and skewed XCI patterns.^121^ However, XCI does not operate in isolation; additional factors influencing gene expression, such as cis-regulatory element variations and tissue-specific transcriptional regulators, must also be considered. Consequently, incorporating multiple genes into XCI analyses improves result accuracy.^97^

With the advent of high-throughput sequencing technologies, RNA sequencing, combined with whole-genome or high-density SNP array data, provides a direct means of quantifying the relative expression of the Xa and Xi. This approach centers on allele-specific expression analysis, which enables precise evaluation of XCI status. Following RNA sequencing, RNA is reverse transcribed into cDNA, and the resulting sequence reads are aligned to a reference genome to identify heterozygous X-linked SNPs. The proportion of reads corresponding to each allele at these SNP loci is then used to calculate the allelic expression ratio.^122^

By integrating high-density SNP data, the parental origin (*i.e.*, paternal *vs*. maternal) of allele-specific expression can be resolved. Comparing the average expression ratio of paternal to maternal alleles allows estimation of the XCI ratio, thereby quantitatively delineating the transcriptional activity of each X chromosome (Fig. 3C).

Advancements in XCI detection and quantification have significantly enhanced our understanding of X-linked gene regulation, phenotypic variation, and disease risk. Methylation-based methods, such as the HUMARA assay and bisulfite sequencing, have long served as foundational tools for XCI analysis, while emerging techniques, such as XCI-ONT, offer improved accuracy and reliability by leveraging long-read sequencing and direct methylation assessment. RNA-based approaches, particularly those incorporating RNA-sequencing and SNP array data, provide complementary insights into X-linked transcriptional dynamics. Collectively, these methodologies are essential for refining XCI profiling, enabling patient stratification in clinical contexts, and facilitating the development of XCI-targeted therapeutic interventions (Table 2).

**Table 2:** Comparative overview of X chromosome inactivation (XCI) detection methods: Core features and trade-offs.

Method	Target	Principle	Advantages	Limitations
HUMARA assay	DNA methylation (*AR* gene)	PCR, methylation-sensitive enzymatic digestion (HpaII), and fragment length analysis	Widely used; high heterozygosity (CAG repeats)	PCR artifacts; fluorescence detection bias; enzyme digestion efficiency dependence
Dual *AR*/*RP2* approach	DNA methylation (*AR* gene and *RP2* gene)	Combines *AR* and *RP2* analysis to improve reliability	Reduces single-locus bias; higher reliability	Increased technical complexity; still PCR-dependent
Bisulfite sequencing	DNA methylation (genome-wide)	Bisulfite converts C to U (unmethylated); methylation-specific sequencing	No enzymes; quantitative	Conversion inefficiency; complex design
XCI-ONT	DNA methylation and repeat length	CRISPR-enriched loci, nanopore sequencing (direct methylation and repeat analysis	No PCR bias; high-resolution data	Expensive; requires a nanopore platform
RNA sequencing and SNP analysis	RNA expression (allele-specific)	RNA sequencing; SNP arrays	Direct transcription insight; tissue-specific	Needs high-quality RNA; No methylation data

Note: HUMARA, human androgen receptor; *AR*, androgen receptor; *RP2*, retinitis pigmentosa 2; SNP, single-nucleotide polymorphism; HpaII, haemophilus parainfluenzae II; ONT, Oxford Nanopore Technologies.

## XCI-based therapeutic strategies for X-linked disorders

XCI leads to monoallelic expression of X-linked genes in female cells. In X-linked disorders, pathogenic mutations on the Xa often result in loss of functional protein, driving disease pathogenesis. This suggests that reactivating the Xi to restore expression of the wild-type allele could be a potential therapeutic approach. The feasibility of this approach has been demonstrated in RTT. In RTT mouse models, reactivation of wild-type *Mecp2* expression, even in adult animals, reverses neurological and behavioral deficits, underscoring the potential for phenotypic rescue post-development.^123^ Thus, targeted reactivation of silenced wild-type alleles on the Xi represents a promising therapeutic avenue for X-linked disorders.

## Inhibitors of X-chromosome inactivation factors (XCIF)

X-chromosome inactivation factors (XCIFs) are essential for selectively silencing X-linked genes. Recent studies have identified two XCIFs, activin A receptor type I (ACVR1) and 3-phosphoinositide-dependent protein kinase 1 (PDPK1), whose inhibition can reactivate the Xi in differentiated mouse embryonic stem cells. ACVR1 likely maintains *Xist* expression by regulating its transcription and chromatin state, while PDPK1 promotes reactivation of silenced genes by modulating *Xist* RNA localization, stability, and chromatin accessibility.

ACVR1 is a component of the bone morphogenetic protein (BMP) signaling pathway. Upon BMP signal activation, phosphorylated Sma- and Mad-related proteins (SMAD) enter the nucleus and regulate transcription. When ACVR1 is blocked by drugs, SMAD proteins cannot be phosphorylated and are unable to enter the nucleus. As a result, SMAD cannot bind to the *Xist* promoter region, leading to the suppression of *Xist* transcription. With the inactivation of *Xist*, the previously silenced Xi-*Mecp2* is gradually derepressed and re-expressed. Mechanistic target of rapamycin kinase (mTOR) and serum/glucocorticoid-regulated kinase 1 (SGK1) are two downstream effectors of PDPK1, both of which belong to the PI3K/AKT signaling pathway. YY1 is a key transcriptional activator for the *Xist* promoter, with multiple binding sites. Following the inhibition of mTOR or SGK1, YY1 fails to accumulate at the *Xist* promoter, resulting in transcriptional suppression. Inhibition of mTOR/SGK1 leads to a reduction in Histone H3 lysine 4 trimethylation (H3K4me3) levels at the *Xist* promoter, thereby decreasing its activity. Similar to ACVR1 inhibitors, both mTOR and SGK1 inhibitors significantly reduce *Xist* transcripts, resulting in the reactivation of Xi-*Mecp2*.

**Figure 4:**
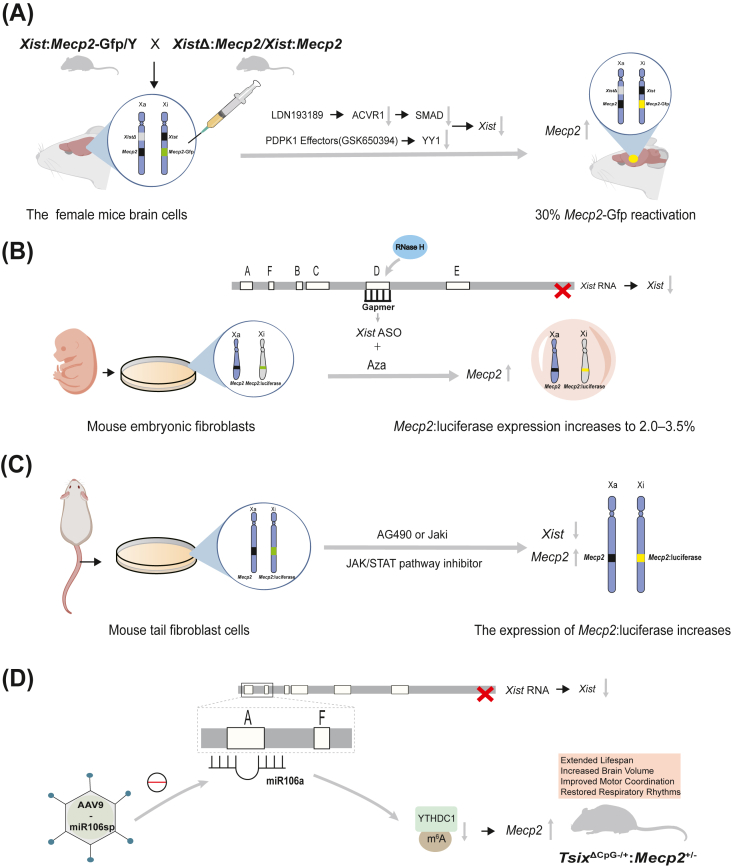
The inactive X chromosome (Xi) reactivation as a therapeutic approach for X-linked disorders. **(A)** Reactivation of Xi-linked methyl-CpG-binding protein 2 (*Mecp2*) in female mouse brain cells using activin A receptor type I (ACVR1) and 3-phosphoinositide-dependent protein kinase 1 (PDPK1) inhibitors. The *Xist*:*Mecp2*-Gfp/Y male mouse was crossed with *Xist*Δ:*Mecp2*/*Xist*:*Mecp2* female mice. This cross generated the *Xist*Δ:*Mecp2*/*Xist*:*Mecp2*-GFP mice, where the active X chromosome (Xa) lacks *Xist* and expresses *Mecp2*, while Xi carries the *Mecp2*-GFP reporter and remains silenced. ACVR1 inhibition: LDN193189 blocks ACVR1, a component of the bone morphogenetic protein (BMP) signaling pathway. When ACVR1 is inhibited, Sma- and Mad-related proteins (SMAD) cannot be phosphorylated, preventing them from entering the nucleus and binding to the *Xist* promoter. PDPK1 inhibition: GSK650394 targets PDPK1, which modulates *Xist* RNA localization, stability, and chromatin accessibility. PDPK1 inhibition reduces the expression of *Xist* by preventing its interaction with the Yin Yang 1 (YY1) transcription factor at the *Xist* promoter. This leads to the reactivation of Xi-linked *Mecp2*. The combined treatment of ACVR1 and PDPK1 inhibitors results in a 30% reactivation of Xi-*Mecp2*-GFP expression in female cortical cells. **(B)** Synergistic reactivation of the X-inactive chromosome via DNA demethylation and *Xist*-targeting antisense oligonucleotides. 5-aza-2-deoxycytidine (Aza), a small-molecule inhibitor of DNA methylation, reduces methylation marks on the *Xist* promoter, facilitating the reactivation process. Gapmers1, a specific type of antisense oligonucleotide (ASO), binds to the Repeat A region of *Xist*, utilizing the RNA degradation mechanism mediated by ribonuclease H (RNase H) to selectively degrade *Xist* RNA, thereby promoting the activation of the *Mecp2* gene on the Xi. In mouse embryonic fibroblasts (MEFs) with a *Mecp2*-luciferase reporter, this combined treatment results in a 2%–5% restoration of normal *Mecp2* levels. **(C)** Reactivation of Xi-linked *Mecp2* in mouse tail fibroblasts using JAK/STAT inhibitors. Transfected mouse tail fibroblasts carrying the *Mecp2*-luciferase reporter gene located on the Xi were treated with AG490 (a JAK2 inhibitor) or Jaki (a pan JAK/STAT pathway inhibitor). These inhibitors suppress the JAK/STAT signaling pathway. Inhibition of JAK/STAT reduces the expression of *Xist*, thereby reactivating the *Mecp2* gene on the Xi. This leads to an increase in *Mecp2*:luciferase expression. **(D)** miR106a reactivates the Xi. In the *Tsix*^ΔCpG−/−^:*Mecp2*^+/−^ mice, the adeno-associated virus serotype 9 (AAV9) vector carrying the miR106a inhibitor miR106a sponge (miR106sp) targets the repeat A region of *Xist* RNA, resulting in decreased *Xist* RNA stability and disruption of N6-methyladenosine (m^6^A) modification and YTH domain-containing 1 (YTHDC1) binding. As a result, the *Mecp2* protein level significantly increases, improving the lifespan, motor coordination, respiratory rhythms, and brain volume in mice.

In female mouse fibroblast cell lines, shRNA-mediated knockdown of ACVR1 or pharmacological inhibition of PDPK1 reactivated Xi-linked genes, including *MECP2*.^124^ Combined treatment with ACVR1 inhibitors and PDPK1 effectors increased *Mecp2* expression and ameliorated morphological deficits in RTT neurons, rescuing somatic cell size and dendritic branching. To precisely quantify Xi-linked *Mecp2* reactivation in the brain, a *Xist*Δ:*Mecp2*/*Xist*:*Mecp2*-GFP mouse model was developed. In this model, the active X chromosome lacks *Xist* and expresses wild-type *Mecp2*, while the inactive X carries a *Mecp2*-GFP fusion reporter (Fig. 4A). Intracerebral injection of the combined inhibitors in female mice resulted in 30% reactivation of Xi-*Mecp2*-GFP in cortical cells.^125^

However, small-molecule inhibitors may broadly reactivate Xi-linked genes rather than selectively targeting specific loci (*e.g.*, *MECP2*), potentially leading to genomic dosage imbalance. Therefore, optimizing inhibitors to selectively target XCI-related factors is crucial for minimizing off-target effects and ensuring therapeutic safety.

## Combination of DNA methylation inhibitors (decitabine [5-aza-2-deoxycytidine, aza]) and antisense oligonucleotides (ASOs)

DNA methylation is a key epigenetic modification that maintains XCI, particularly through promoter hypermethylation that enforces gene silencing. ASOs are short synthetic nucleic acid sequences designed to bind specific RNA molecules and disrupt their function. In this context, *Xis*t-targeting ASOs bind to *Xist* RNA, blocking its ability to recruit chromatin modifiers and sustain XCI. The combination of DNA methylation inhibitors (*e.g.*, Aza) and *Xist-*targeting ASOs has been explored as a potential approach to reactivate Xi.

In proof-of-concept experiments, mouse embryonic fibroblasts (MEFs) transfected with a *Mecp2*-luciferase reporter were treated with *Xist* ASOs and Aza. After 5 days, luciferase activity increased by 2.0%–3.5%, reflecting partial reactivation of the Xi-linked *MECP2* allele. Notably, the combined treatment achieved a 30,000-fold up-regulation of *MECP2* expression from the Xi, equating to 2%–5% of normal *MECP2* levels (Fig. 4B). Despite these results demonstrating the feasibility of Xi reactivation, several challenges remain. The modest reactivation efficiency (2%–5%) may be insufficient for clinical benefit, and non-specific effects of Aza (*e.g.*, genome-wide DNA hypomethylation) pose potential safety concerns. Further refinement of ASO specificity and localized delivery methods (*e.g.*, nanoparticles) could enhance therapeutic efficacy while minimizing off-target effects.^126^

While Aza (decitabine) is FDA-approved for treating acute myeloid leukemia, its systemic toxicity (*e.g.*, myelosuppression) limits its suitability for chronic neurological disorders. Although short-term pulsed regimens may reduce toxicity, the necessity for prolonged, repeated treatments to sustain Xi reactivation remains unclear and warrants further investigation. Additionally, *Xist*-targeting ASOs may inadvertently affect non-*Xist* RNAs or disrupt broader epigenetic regulation. Advanced delivery strategies (*e.g.*, lipid nanoparticles) could enhance ASO bioavailability and neural tissue selectivity, thereby reducing systemic exposure and off-target activity.

## JAK/STAT pathway inhibitors

The epidermal growth factor receptor (EGFR) exhibits inhibitory activity in certain signaling contexts. A high-throughput luciferase assay in mouse fibroblasts harboring an inactive *MeCP2*-luciferase reporter identified Jaki, a pan-JAK/STAT inhibitor, as a potential Xi reactivator. This screening also revealed that AG490 (a JAK2 kinase inhibitor) and Jaki can reactivate *Mecp2* by suppressing the JAK/STAT pathway, particularly through *Xist* and *Rnf12* down-regulation. The luminescence-based high-throughput screening revealed that a twofold or greater increase in relative luminescence units (RLU) indicates the activation of *Mecp2*. However, in human-hamster hybrid cell lines, AG490 showed weaker *Mecp2* reactivation compared with the robust effects of 5-Aza, suggesting that the effectiveness of JAK/STAT inhibitors may depend on cell type or species-specific regulatory contexts (Fig. 4C).^127^

## Epigenetic editing

Beyond pharmacological inhibition, epigenetic editing has emerged as a precise strategy for Xi reactivation. Song Lou et al conducted a genome-wide CRISPR/Cas9 loss-of-function screen in female mouse fibroblasts and identified several microRNAs (miRNAs) as regulators of XCI, among which miR106a emerged as the most critical. Inhibition of miR106a led to the reactivation of multiple genes on the Xi, including *Mecp2*, glucose-6-phosphate dehydrogenase X-linked (*G6pdx*), lysosomal-associated membrane protein 2 (*Lamp2*), and phosphoglycerate kinase 1 (*Pgk1*). Mechanistically, miR106a exerts its function by directly binding to the Repeat A region of *Xist,* thereby stabilizing *Xist* RNA and supporting its role in maintaining XCI. Inhibition of miR106a also disrupts m^6^A modification and YTHDC1 binding, resulting in abnormal expression and localization of *Xist*, thus impairing the balance of XCI. In a Rett syndrome mouse model (*Tsix*^ΔCpG−/+^; *Mecp2*^+/−^ females), delivery of a miR106a inhibitor, miR106a sponge (miR106sp), via an adeno-associated virus serotype 9 (AAV9) vector significantly increased *Mecp2* expression in the brain (restored to ∼32% of wild-type levels) and ameliorated multiple pathological phenotypes, including reduced lifespan, motor coordination deficits, abnormal respiratory rhythms, and reduced brain volume (Fig. 4D).^128^ This study highlights the role of miR106a in X chromosome inactivation, but the exact mechanism by which it affects Xi transcription remains unclear. While miR106a is thought to regulate *Xist* conformational changes and interactions, further research is needed. This could potentially serve as a therapeutic approach for other X-linked diseases.

A recent study employed dCas9-Tet1 (a DNA demethylation tool) and dCpf1-CTCF (a chromatin insulator tool) to demethylate the *MECP2* promoter, successfully reactivating Xi-linked *MECP2* in RTT human embryonic stem cells (hESCs). Notably, neurons derived from methylation-edited RTT hESCs exhibited rescued cellular morphology and electrophysiological deficits, demonstrating functional recovery.^129^ Future studies are needed to evaluate the effects of this epigenome editing approach in animal models of RTT, particularly regarding its impact on behavioral outcomes.

In addition, reactivation of the silent wild-type *ALAS2* allele has been achieved in XLSA by using Aza in mutated iPSC-derived hematopoietic progenitor cells.^130^ In neurodegeneration with brain iron accumulation, the normal WD Repeat domain 45 (*WDR45*) allele was activated in patient-derived fibroblasts through biotin supplementation.^131^ Recent studies have also demonstrated that down-regulation of *Rnf12* can reduce *Xist* levels,^132^ which may present a potential alternative approach for future activation of Xi.

However, a major challenge in Xi reactivation is maintaining dosage balance to preserve cellular homeostasis. For example, genetic ablation of *Ftx* in mice reduces *Xist* RNA levels but leads to immune hyperactivation, predisposing animals to autoimmune disorders.^133^ Similarly, conditional deletion of *Xist* in murine hematopoietic compartments induces aggressive myeloproliferative neoplasms and myelodysplastic syndromes, underscoring the tumor-suppressive role of XCI in maintaining genomic stability.^134^

These findings illustrate the dual-edged nature of Xi reactivation strategies. While restoring wild-type gene expression (*e.g.*, *MECP2*) may alleviate disease-specific deficits, unintended off-target reactivation of other Xi-linked loci could lead to deleterious consequences. For example, unsilenced oncogenes or immune regulators on the Xi might promote malignancy or autoimmunity. Therefore, achieving locus-specific reactivation, rather than global Xi derepression, is paramount to ensure therapeutic safety.

Over 20% of human X-linked genes can escape XCI, meaning they bypass the multiple layers of epigenetic silencing established during XCI and maintain transcriptional activity on the otherwise silent Xi. This results in biallelic expression from both X chromosomes in females.^135^^,^^136^ *MECP2* is a facultative escape gene, meaning it is initially silenced during development but can later be reactivated, demonstrating the potential for gene reactivation. Additionally, constitutive escape genes have been identified, which are expressed across most cell types, individuals, and developmental stages. These genes seem to avoid XCI from the outset, showing little to no silencing during XCI.^137^ Understanding the escape characteristics of different genes is therefore crucial for developing effective strategies to treat X-linked diseases.

## Conclusions and perspectives

Skewed XCI can result in the overexpression or silencing of pathogenic alleles, significantly influencing disease severity. Recent studies have shown a strong correlation between XCI skewing and various X-linked phenotypes, with over 80% of findings linking disease severity to XCI skewness. However, diseases like RTT, Fabry disease, and Duchenne muscular dystrophy, despite receiving substantial attention from researchers, have yet to reach a unified conclusion. This is primarily due to differences in the cohorts studied, including variations in age, mutation sites, and tissue types, as well as inconsistencies in phenotypic assessment criteria, leading to considerable discrepancies in the results. To reliably assess the impact of XCI skewing on phenotypic variability, future studies should analyze familial cohorts with identical mutations, employ standardized phenotyping, and integrate analyses of tissue specificity, age-related dynamics, and other epigenetic modifiers.

Targeting *Xist* RNA to reactivate the inactive X chromosome has shown promising improvements in disease severity in mouse models. Consequently, strategies aimed at reactivating the Xi to restore wild-type gene expression have emerged as a potential approach for treating X-linked diseases. Current therapeutic methods include XCIF inhibitors (*e.g.*, targeting ACVR1/PDPK1), DNA methylation inhibitors (*e.g.*, Aza), ASOs, JAK/STAT pathway inhibitors (*e.g.*, AG490), and epigenetic editing techniques (*e.g.*, miR106a inhibition or dCas9-Tet1). However, each method carries its own limitations, such as non-specific activation and safety concerns. In contrast, epigenetic editing offers more precise targeting of regulatory elements, providing sustained and efficient phenotypic rescue with superior locus specificity, which is crucial for maintaining genomic dosage balance.

Furthermore, precise quantification of XCI skewing is critical for assessing disease risk and evaluating therapeutic efficacy. Traditional DNA methylation detection methods, owing to their technical maturity and operational simplicity, are widely used in initial clinical screening and large-scale cohort studies. Compared with traditional methods, XCI-ONT represents a future direction for more accurate determination of XCI skewness. However, for potential clinical adoption, challenges such as cost reduction and technology standardization need to be addressed.

Research into the mechanisms of XCI is essential for developing innovative therapeutic strategies, with current efforts primarily concentrating on elucidating the role of the *Xist/XIST*. In humans and mice, it is currently believed that *Xist/XIST* RNA recruits chromatin modifiers and gene-silencing factors through its Repeat modules (*e.g.*, A, B, C, D, E) to mediate chromatin remodeling and transcriptional repression on the Xi. However, emerging evidence suggests that the mechanisms in humans differ from those in mice, particularly in the initiation of XCI, the composition of *Xist* Repeat elements, and the regulation by non-coding RNAs. A deeper understanding of the structural features and regulatory mechanisms of *Xist/XIST* RNA in both mice and humans is essential for advancing future therapeutic strategies.

## CRediT authorship contribution statement

**Yuan Fu:** Writing – original draft. **Xuling Tan:** Writing – review & editing. **Lixia Qin:** Writing – review & editing. **Chunyu Wang:** Writing – review & editing, Supervision.

## Funding

This review was supported by the 10.13039/501100001809National Natural Science Foundation of China (No. 82471277) and the 10.13039/501100004735Natural Science Foundation of Hunan Province, China (No. 2024JJ5471).

## Conflict of interests

The authors declared no conflict of interests.
